# Circular RNA CCDC66 promotes gastric cancer progression by regulating c-Myc and TGF-β signaling pathways

**DOI:** 10.7150/jca.37718

**Published:** 2020-02-20

**Authors:** Guifang Xu, Yanke Chen, Min Fu, Xueyan Zang, Mingming Cang, Yanlong Niu, Weiya Zhang, Yu Zhang, Zheying Mao, Meng Shao, Hui Qian, Wenrong Xu, Hui Cai, Pengcheng Jiang, Xu Zhang

**Affiliations:** 1 Institute of Digestive Diseases, The Affiliated People's Hospital of Jiangsu University, Zhenjiang, Jiangsu 212002, China; 2 Jiangsu Key Laboratory of Medical Science and Laboratory Medicine, School of Medicine, Jiangsu University, Zhenjiang, Jiangsu 212013, China; 3 Key Laboratory of Molecular Diagnostics and Precision Medicine for Surgical Oncology in Gansu Province, Gansu Provincial Hospital, Gansu 730000, China

**Keywords:** circular RNA, gastric cancer, circCCDC66, EMT, progression

## Abstract

**Background:** CircRNAs play important roles in cancer development and progression and have the potential to serve as cancer biomarkers. The aim of this study was to investigate the role of circular RNA CCDC66 (circCCDC66) in gastric cancer and to reveal the underlying mechanisms.

**Methods:** The expression of circCCDC66 in GC tissues and cell lines was examined by qRT-PCR. The correlation between circCCDC66 expression level and clinicopathological characteristics was analyzed. The biological roles of circCCDC66 in GC cell apoptosis, proliferation, migration and invasion were determined by flow cytometry, cell counting, cell colony formation, wound healing, transwell migration and matrigel invasion assays. The role of circCCDC66 in GC growth was further confirmed by mouse xenograft tumor model. Western blot and qRT-PCR were used to explore the effects of circCCDC66 on epithelial-mesenchymal transition (EMT)-related gene and protein expression.

**Results:** CircCCDC66 expression was elevated in both GC tissues and cell lines compared to adjacent normal tissues and normal gastric epithelial cell line. The upregulation of circCCDC66 in GC tissues was related to tumor stage and lymphatic metastasis. CircCCDC66 knockdown significantly inhibited GC cell proliferation, migration and invasion and induced cell apoptosis in GC cells. On the contrary, circCCDC66 overexpression had the opposite effects. In addition, circCCDC66 knockdown suppressed the tumorigenesis of GC cells in nude mice. Furthermore, circCCDC66 knockdown inhibited the activation of c-Myc and TGF-β signaling pathways and reversed EMT in GC cells. c-Myc and TGF-β interference blocked circCCDC66-mediated promotion of gastric cancer cell proliferation, migration and invasion.

**Conclusion:** CircCCDC66 promotes GC growth and metastasis by activating c-Myc and TGF-β signaling pathways, suggesting that it may serve as a potential biomarker for GC.

## Introduction

Gastric cancer (GC) is one of the most common cancers worldwide with high morbidity and mortality 1,2. Although many advances have been made in the diagnosis and treatment of this disease, the prognosis of GC patients remains poor with a 5-year overall survival less than 30% in most countries 3. Therefore, there is an urgent need to further understand the molecular mechanism for GC development and progression and to seek for biomarkers for GC early diagnosis and prognosis prediction.

Circular RNAs (circRNAs) are noncoding RNAs that are covalently linked to form a closed circular structure without 5' caps and 3' tails 4,5. CircRNAs display cell or tissue specific expression and are conserved across species due to their resistance to RNase R. Compared to their linear counterparts, circRNAs are highly stable and accumulate in both nucleus and cytoplasm, indicating important roles in human health. CircRNAs participate in a wide range of biological processes, including transcription 6, mRNA splicing 7, RNA decay and translation 8, and their dysregulation leads to abnormal cellular functions and human diseases, such as cardiovascular diseases 9, temporal lobe epilepsy 10, Alzheimer's disease 11, metabolic diseases 12, and cancer 13. Emerging evidence suggest that some circRNAs act as miRNA sponges 14,15 and interact with RNA binding proteins (RBPs) 16,17 to modulate gene expression. For instance, ciRS-7 serves as miR-7 sponge to regulate the expression of several oncogenes 18 and circHIPK3 as miR-124 sponge to suppress cell proliferation in multiple cancers 19.

Recent studies suggest that circRNAs are deregulated in various cancers, including hepatocellular cancer (HCC) 20, colorectal cancer (CRC) 21, esophageal squamous cancer 22, and breast cancer 23. CircRNAs are involved in cancer growth, metastasis, and therapy resistance 24,25. CircRNAs have also been shown to play important roles in gastric cancer proliferation, migration and metastasis and can serve as diagnostic biomarkers 26. Zhang *et al.* demonstrate that the expression of circLARP4 is downregulated in GC tissues and circLARP4 represents an independent prognostic factor for overall survival of GC patients 27. Liu *et al.* demonstrate that circYAP1 functions as a tumor suppressor in GC cells by targeting the miR-367-5p/p27 axis and it provides a prognostic indicator of survival in GC patients 28. Yang *et al.* demonstrate that circ-CTNNB1 activates β-catenin signaling to promote gastric cancer progression through DDX3-mediated transactivation of YY1 29. These findings suggest that circRNAs are crucial for gastric cancer development and progression and have great promise to be used as biomarkers and targets for gastric cancer diagnosis and therapy.

Circular RNA CCDC66 (circCCDC66) was previously found to be highly expressed in human colorectal cancer and could promote CRC growth and metastasis 21. However, its role in gastric cancer has not been reported yet. In this study, we found that the expression of circCCDC66 was increased in gastric cancer tissues and cell lines, and its expression level was associated with tumor stage and lymphatic metastasis. CircCCDC66 knockdown inhibited while circCCDC66 overexpression promoted GC growth and metastasis by inducing epithelial to mesenchymal transition (EMT) through the activation of c-Myc and TGF-β signaling pathways.

## Materials and Methods

### Patients and samples

A total of 70 patients with gastric cancer who underwent gastrectomy at the First People's Hospital of Zhenjiang between November 2015 and October 2017 were enrolled in this study. The adjacent non-cancerous tissues were collected at 5 cm away from the tumor site. Tumor stages were classified following the 8^th^ edition tumor-node-metastasis (TNM) classification system of the American Joint Committee on Cancer. Histological grade was assessed by two experienced pathologists. All the patients enrolled in this study had not received any radiation or chemotherapy and had no other cancers before. This study was approved by Institutional Reviewing Board of First People's Hospital of Zhenjiang and informed consent was obtained from each patient.

### Primer design and synthesis

The primer for circCCDC66 and small molecular RNA U6 was synthesized by Hanbio Biotechnology (Shanghai, China). The sequences of circCCDC66 and U6 were as follows: circCCDC66: F: ACCTACAACCGGAAGCCAG, R: AGCAGTACTGTTTCCTGATGC; U6: F: CTCGCTTCGGCAGCACA, R: AACGCTTCACGAATTTGCGT.

### Quantitative real-time polymerase chain reaction (qRT-PCR)

Total RNA was extracted from tissues and cells by Trizol reagent (Invitrogen) and quantified by NanoDrop Spectrophotometer. Total RNA was reverse transcribed to cDNA by using HiScript 1st Strand cDNA Synthesis Kit (Vazyme, Nanjing, China). The cDNA samples were quantified on CFX96 Real-time PCR Detection System (Bio-Rad, Hercules, CA, USA) by using UltraSYBR Mixture (CWBIO, Beijing, China) according to the manufacturer's instructions. U6 was used as internal control.

### Cell culture

Human gastric cancer cell lines BGC-823, MGC-803, SGC-7901, and HGC-27 were purchased from the Shanghai Institute of Biochemistry and Cell Biology, Chinese Academy of Sciences (Shanghai, China). Human normal gastric epithelial cell line GES-1 was obtained from Gefan Biological Technology (Shanghai, China). BGC-823, SGC-7901, HGC-27, and GES-1 cells were routinely cultured in RPMI 1640 medium (Invitrogen, Carlsbad, CA, USA) supplemented with 10% fetal bovine serum (FBS) (Gibco). MGC-803 cells were cultured with high glucose DMEM (Invitrogen). All the cell lines were cultured at 37**^o^**C humidified atmosphere containing 5% CO_2_.

### Cell transfection

Control and circCCDC66 siRNAs were synthesized by Hanbio Biotechnology (Shanghai, China). The sequences of siRNAs were as follows: si-circCCDC66: 5'-CAAUUAGAGCAUCAGGAAATT-3'; si-control: 5'-UUCUCCGAACGUGUCACGUTT-3'; si-TGF-β1: 5'-GCAGAGUACACACAGCAUATT-3'; si-c-Myc: GUGCAGCCGUAUUUCUACUTT. GC cells (2×10^5^/well) in logarithmic growth period were transfected with overexpressing plasmid and silencing siRNAs by using LipoFiter (Hanbio, Shanghai, China) according to the manufacturer's instructions. Cells were collected at 36 hours after transfection. The transfection efficiency was assessed by qRT- PCR.

### Cell counting assay and colony formation assay

At 24 hours after transfection, the cells were collected and plated into 24-well plates at 10^4^ cells/well. The number of cells were counted every day for consecutive 6 days. For colony formation assay, the transfected cells (10^3^ cells/well) were plated into the 6-well plates and cultured for consecutive 10 days. The colonies were fixed with polyformaldehyde and stained with 0.1% crystal violet.

### Cell migration and invasion assays

For cell migration assay, the transfected cells (2×10^4^ cells/well) were suspended in serum-free medium and plated on the upper chamber of transwell with 8 μm size pore (Corning, NY, USA). For cell invasion assay, the transfected cells (1×10^5^cells/well) were placed in the upper chamber of transwell with 50 μl matrigel coated. The lower chamber of transwell was filled with 600 μl RPMI 1640 medium containing 10% FBS. After incubation for 36 h, the cells on the surface of upper chamber were removed by scraping with a cotton swab, while the cells on the lower surface of the inserts were fixed with polyformaldehyde for 30 min and stained with 0.1% crystal violet for 15 min. After that, each insert was counted under an inverted microscope (Olympus, Tokyo, Japan).

### Wound healing assay

The transfected cells were plated in 6-well plates (4×10^5^ cells /well). Wounds were created by using a 100 μl pipette tip at 24 hours after inoculation. Then, the floating cells and debris were washed out by PBS. Wound healing was observed at different time points and the control group and experimental group were photographed at the same time.

### Cell apoptosis assay

Cells were harvested at 36 hours after transfection and then stained with Annexin Alexa Fluor 647 and PI at room temperature in dark for 15 min. FACSCalibur flow cytometry (BD Biosciences, San Jose, CA, USA) was used to evaluate the percentage of apoptotic cells. Finally, Cell Quest Software was used to analyze the data.

### Western Blot

Proteins were extracted with Radio-Immunoprecipitation Assay (RIPA) extraction reagent (Beyotime, Beijing, China). Equal amounts of protein were separated via 12% polyacrylamide gel, then switched to PVDF membrane (Millipore, Billerica, MA, USA). After being blocked with 5% non-fat milk for 1 h, the PVDF membranes were incubated with specific primary antibodies (Cell Signaling Technology, Shanghai, China) including p21, poly(ADP-ribose) polymerase (PARP), caspase-3, Bcl-2, E-cadherin, N-cadherin, Slug, Vimentin, c-Myc, TGF-β, p-SMAD2, SMAD2 and MMP9 antibodies. GAPDH (Sigma-Aldrich, St. Louis, MO, USA) was used as the internal control. Afterwards, the membrane was incubated with secondary antibodies (CST) for 2 h. Finally, protein bands were visualized by using chemiluminescence (Millipore).

### Mouse tumor model

4-6 weeks old male athymic nude mouse were purchased from Nanjing Model Animal Center (Nanjing, China) and maintained in SPF condition with a standard 12-h light-dark cycle. SGC-7901 cells transfected with si-circCCDC66 and si-control were suspended in PBS (6×10^6^/200 μl) and then subcutaneously injected into the mice. Tumor growth was monitored twice per week. Tumor volume was evaluated using the formula 0.5× (a×b^2^), in which a means the long diameter, b represents the short diameter. Four weeks later, the mice were sacrificed and the tumor tissues were harvested. The tumor tissues were weighed and fixed in 4% formalin for immunohistochemical analysis.

### Immunohistochemistry

Tumor tissue sections were incubated with primary monoclonal antibody against Ki-67 followed by incubation with the secondary antibody for 30 min at room temperature. Afterwards, the sections were incubated with 3, 3′-Diaminobenzidine (3, 3′-DAB, Maxim, Fuzhou, China) for 5 min, then counterstained with hematoxylin for 30 s. Finally, the sections were photographed under a TE2000 microscope (Nikon, Tokyo, Japan).

### Statistical analysis

All the data were expressed as mean ±SD. Chi-square test or one-way analysis of variance (ANOVA) were conducted by using SPSS20.0 software (SPSS, Chicago, IL, USA) to compare the statistical differences. The results were considered statistically significant when *P*-value<0.05.

## Results

### Circ CCDC66 is upregulated in gastric cancer tissues and cell lines

**Figure 1:**
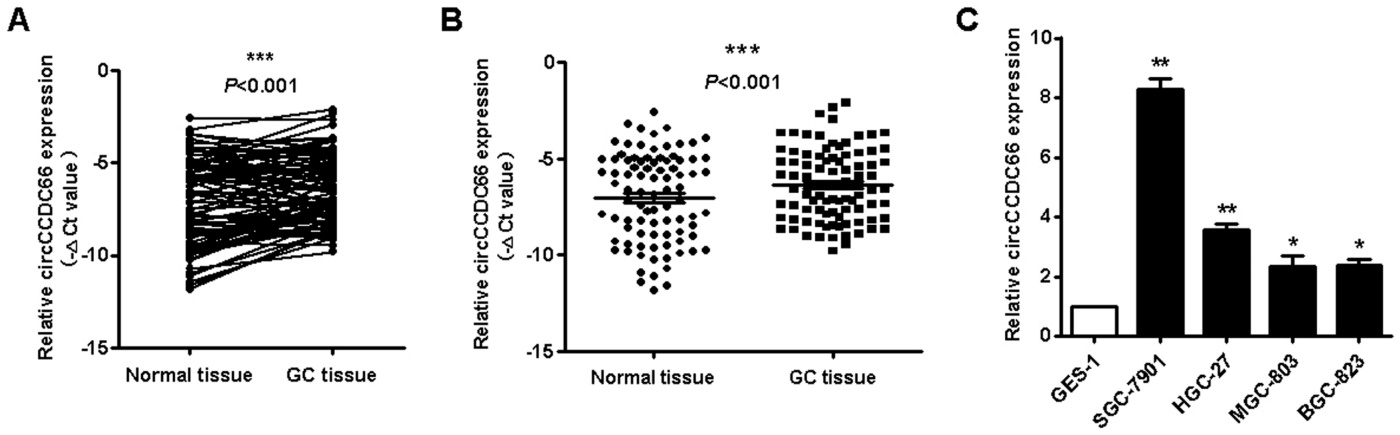
CircCCDC66 is upregulated in gastric cancer tissues and cell lines. (**A** and **B**) The expression levels of circCCDC66 in GC tissues and paired normal tissues (n=70). (**C**) The expression profiles of circCCDC66 in SGC-7901, HGC-27, MGC-803, BGC-823 and GES-1 cell lines. The experiment was repeated for three times. *P<0.05, **P <0.01, ***P <0.001.

We detected the expression of circCCDC66 in gastric cancer tissues and cell lines by using qRT-PCR. As shown in Figures 1A and 1B, circCCDC66 was overexpressed in 68.5% (48/70) of GC tissues compared to adjacent normal tissues (*P*<0.001). We then analyzed the correlation between circCCDC66 expression level and clinicopathological parameters of GC patients. CircCCDC66 expression level was positively associated with tumor stage (*P*=0.01) and lymphatic metastasis (*P*=0.014) (Supplementary Table 1). Furthermore, circCCDC66 expression was also upregulated in GC cell lines compared to normal gastric epithelial cell line (Figure 1C). In summary, circCCDC66 was upregulated in GC tissues and cell lines and its expression level was associated with GC progression.

### CircCCDC66 knockdown inhibits and circCCDC66 overexpression promotes the proliferation of gastric cancer cells *in vitro*

**Figure 2:**
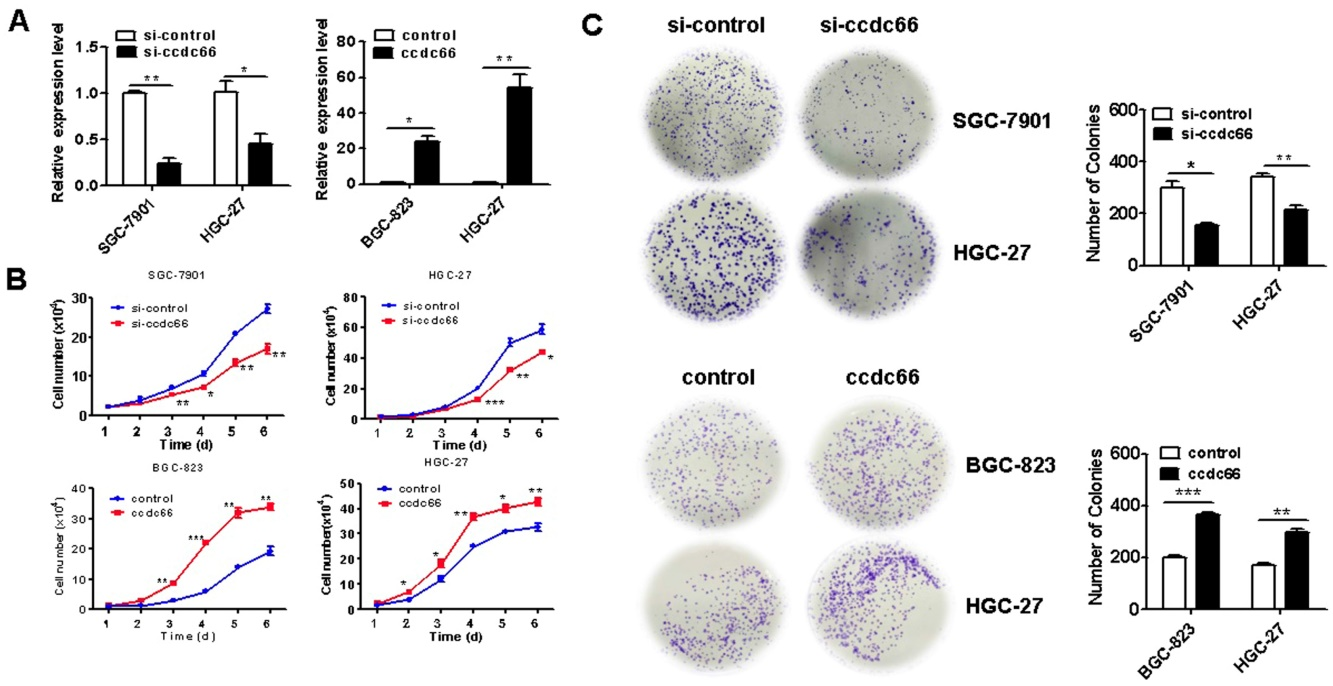
CircCCDC66 knockdown inhibits while overexpression promotes the proliferation of gastric cancer cells. (**A**) CircCCDC66 expression levels in siRNAs and overexpressing plasmid transfected GC cells. (**B**) Cell growth curves for circCCDC66 siRNAs and overexpressing plasmid transfected GC cells. (**C**) The colony formation ability of circCCDC66 siRNAs and overexpressing plasmid transfected GC cells. *P < 0.05, **P < 0.01, ***P < 0.001.

To determine the role of circCCDC66 in GC cell proliferation, we knocked down circCCDC66 in SGC-7901 and HGC-27 cells and overexpressed circCCDC66 in BGC-823 and HGC-27 cells by gene transfection. The efficiency of gene knockdown and gene overexpression was confirmed by qRT-PCR (Figure 2A). The results of cell counting assay showed that the number of cells in si-circCCDC66 group was less than that in si-control group at 48 hours after cell plating (Figure 2B). In addition, the number of cell colonies in si-circCCDC66 group was also significantly less than that in si-control group (Figure 2C). On the contrary, the number of cells and cell colonies in circCCDC66 overexpressing group was significantly more than that in control group (Figures 2B and 2C).

### CircCCDC66 silencing inhibits gastric cancer growth* in vivo*

**Figure 3:**
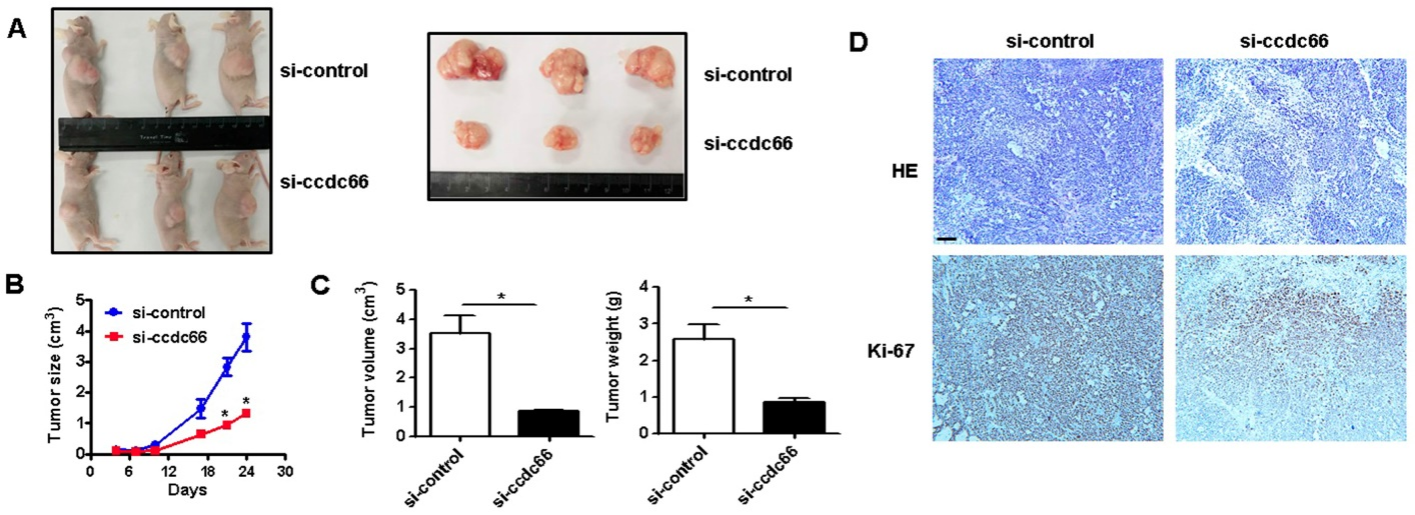
circCCDC66 knockdown inhibits gastric cancer growth *in vivo.* (**A**) Representative images of tumor-bearing mice and tumors in control and circCCDC66 knockdown groups. (**B**) Tumor growth curves of mice in control and circCCDC66 knockdown groups (n=3). (**C**) Tumor volumes and weights of mice in control and circCCDC66 knockdown groups (n=3). (**D**) HE staining and immunohistochemical staining of Ki- 67 in mouse tumor tissues in control and circCCDC66 knockdown groups. Scale bar: 100 μm. *P < 0.05, **P < 0.01, ***P < 0.001.

We next wanted to know the role of circCCDC66 in GC growth *in vivo*. Athymic nude mouse were injected with si-control and si-circCCDC66 transfected SGC-7901 cells subcutaneously. As shown in Figures 3A and 3B, the tumors in si-circCCDC66 group grew slowly compared to those in si-control group. At the end of experiment, the mean volume and weight of tumors in si-circCCDC66 group were smaller than that in si-control group (Figure 3C). The results of immunohistochemical analysis showed that tumor tissues in si-circCCDC66 group had less Ki-67 positive cells than that in si-control group (Figure 3D). These data suggest that circCCDC66 could promote gastric cancer growth *in vivo*.

### CircCCDC66 gene silencing induces cell apoptosis

**Figure 4:**
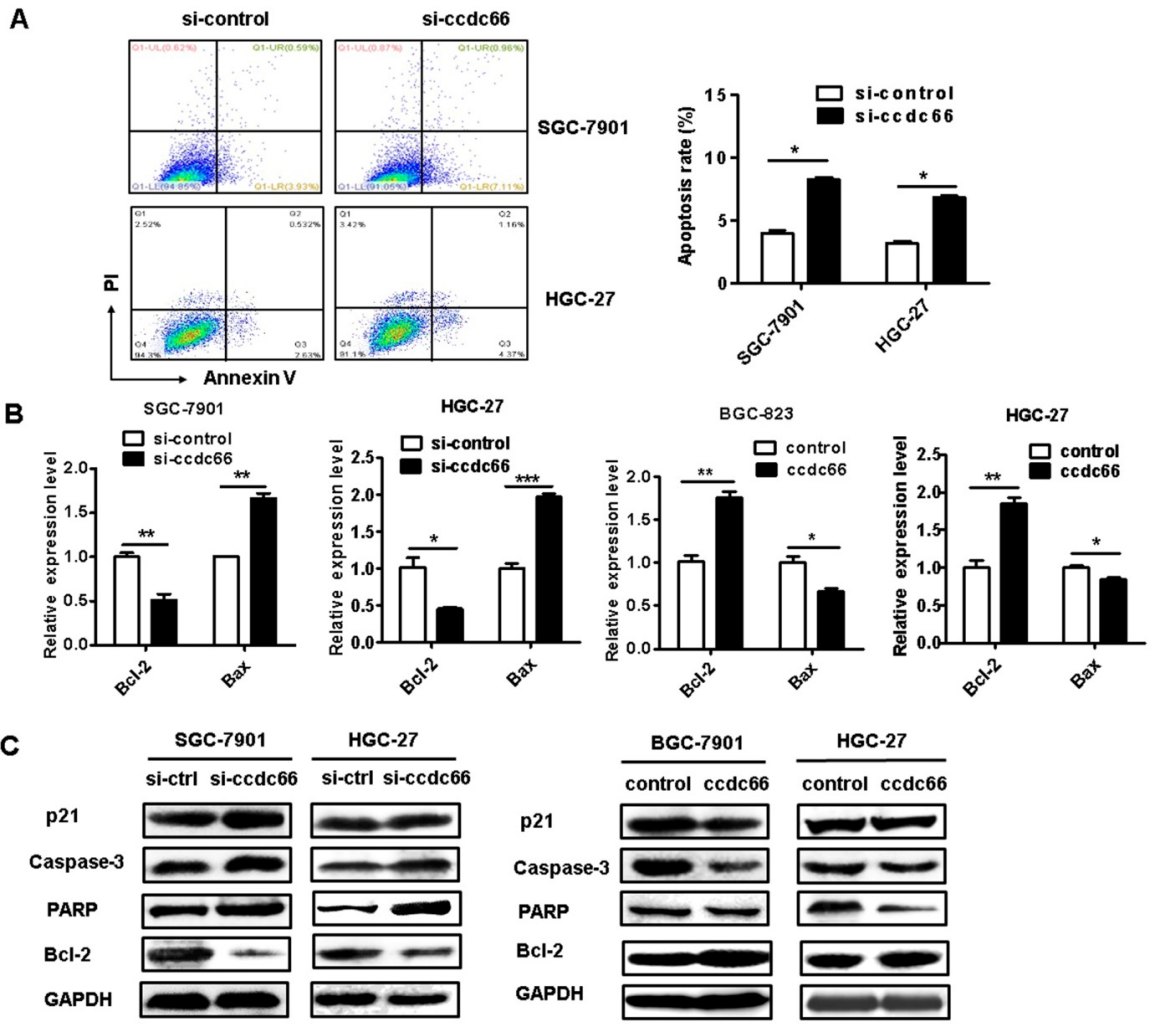
CircCCDC66 gene silencing suppresses while gene overexpression promotes the apoptosis of GC cells. (**A**) Flow cytometric analyses of cell apoptosis in control and circCCDC66 knockdown groups. (**B**) QRT-PCR analyses of Bcl-2 and Bax gene expression in circCCDC66 knockdown and overexpressing GC cells. (**C**) Western blot analyses of p21, poly (ADP- ribose) polymerase (PARP), Bcl-2 and Caspase-3 expression in circCCDC66 knockdown and overexpressing GC cells. All the experiments were repeated for three times. *P <0.05, **P <0.01, ***P <0.001.

We further determined the effect of circCCDC66 on gastric cancer cell apoptosis. The results of flow cytometric analyses indicated that circCCDC66 knockdown promoted cell apoptosis in both SGC-7901 and HGC-27 cells (Figure 4A). Moreover, the results of qRT-PCR showed that circCCDC66 silencing inhibited the expression of Bcl-2 but promoted the expression of Bax. The expression of Bcl-2 was upregulated and that of Bax downregulated in circCCDC66 overexpressing GC cells (Figure 4B). The results of western blot indicated that circCCDC66 knockdown promoted the expression of p21, cleaved PARP and caspase-3 while inhibited that of Bcl-2. By contrast, circCCDC66 overexpression showed inverse changes in p21, cleaved PARP and Caspase-3, and Bcl-2 expression in GC cells (Figure 4C). The results suggest that circCCDC66 could inhibit GC cell apoptosis.

### CircCCDC66 knockdown inhibits while gene overexpression promotes GC cell migration and invasion

**Figure 5:**
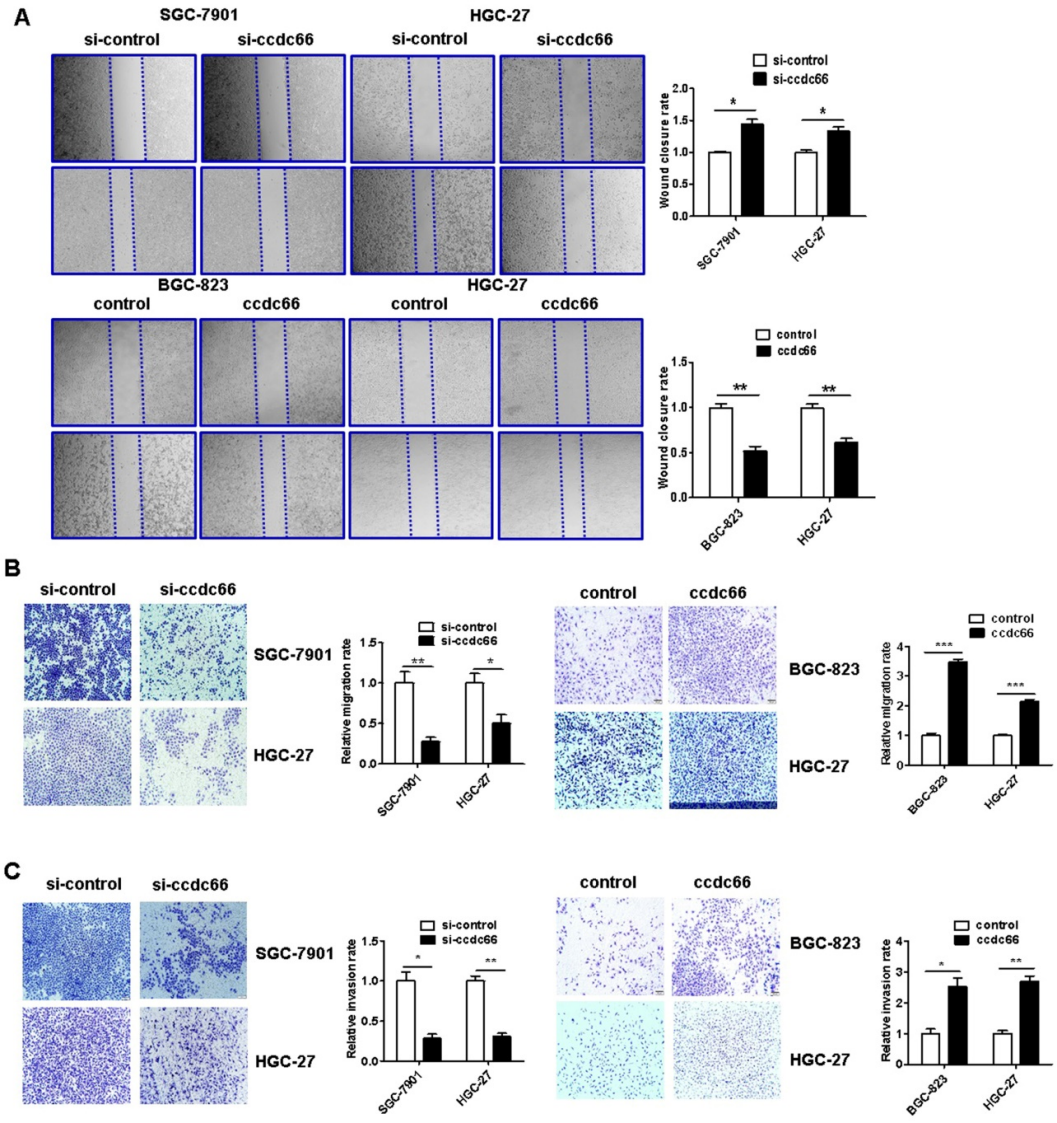
CircCCDC66 knockdown inhibits while overexpression promotes the migration and invasion of gastric cancer cells. (**A**) Wound healing assays for circCCDC66 siRNAs and overexpressing plasmid transfected GC cells. (**B**) Transwell migration assays for circCCDC66 siRNAs and overexpressing plasmid transfected GC cells. (**C**) Matrigel invasion assays for the invasive abilities of GC cells transfected with circCCDC66 siRNAs and overexpressing plasmid. All the experiments were performed in triplicate. *P < 0.05, **P < 0.01, ***P < 0.001.

The results of wound healing assay showed that circCCDC66 knockdown caused a notably lower scratch closure rate than that observed in control cells. CircCCDC66 overexpressing cells showed a higher scratch closure rate than control cells (Figure 5A). The results of transwell migration and matrigel invasion assays showed that circCCDC66 knockdown suppressed while circCCDC66 overexpression enhanced the migration and invasion abilities of GC cells (Figure 5B and 5C).

### CircCCDC66 regulates c-Myc and TGF-β signaling pathways in GC cells

**Figure 6:**
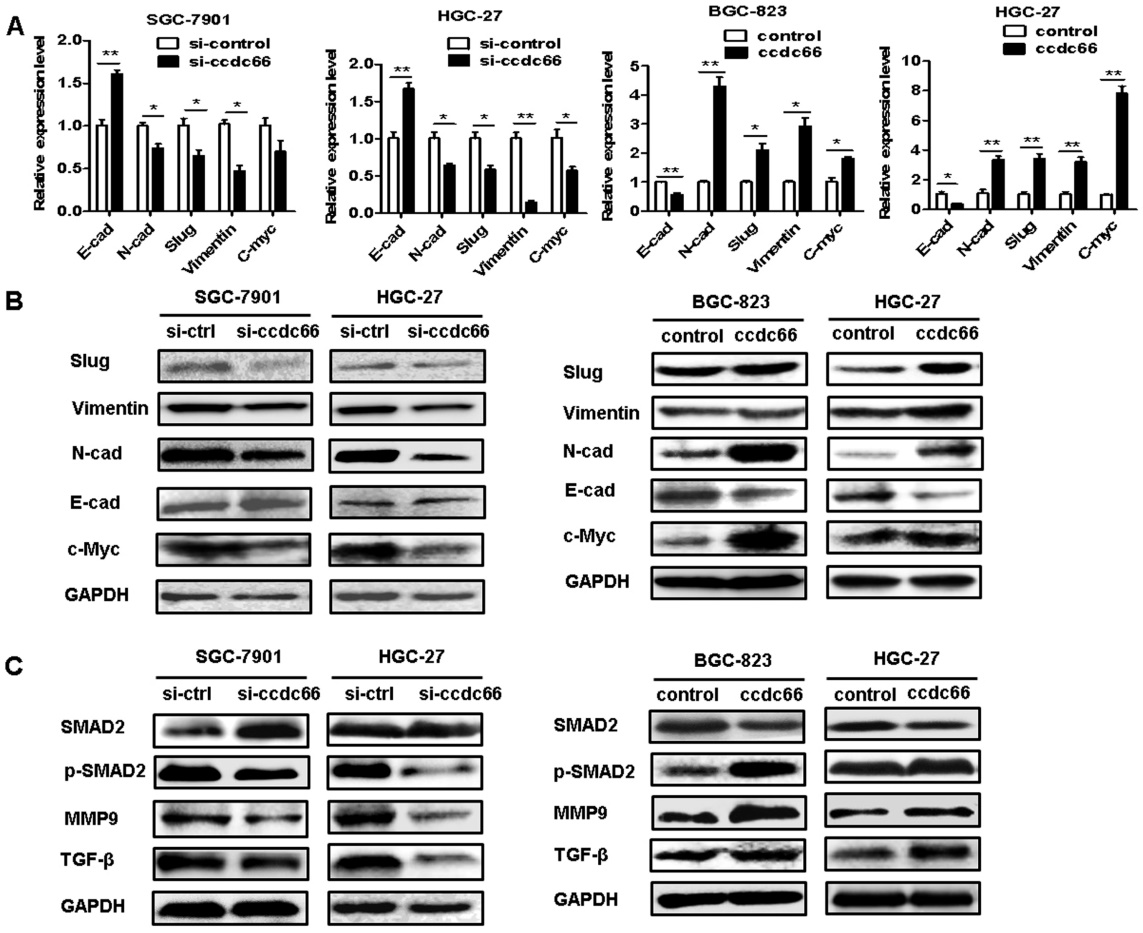
CircCCDC66 regulates c-Myc and TGF-β signaling pathways in GC cells. (**A**) QRT- PCR and (**B**) Western blot analyses of EMT-specific markers in GC cells transfected with circCCDC66 siRNAs and overexpressing plasmid. (**C**) Western blot analysis of TGF-β, SMAD2, p-SMAD2 and MMP9 in GC cells transfected with circCCDC66 siRNAs and overexpressing plasmid. All the experiments were repeated for three times. *P<0.05, **P <0.01, ***P <0.001.

Since circCCDC66 has been shown to affect cancer cell proliferation, migration and invasion and it has been reported to regulate several oncogenic genes including c-Myc, we performed qRT-PCR and western blot to explore the effect of circCCDC66 on the expression of c-Myc and TGF-β signaling pathways in GC cells. As shown in Figure 6A, circCCDC66 knockdown decreased the expression of N-cadherin, Vimentin, Slug and c-Myc but increased the expression of E-cadherin in SGC-7901 and HGC-27 cells. CircCCDC66 overexpression enhanced the expression of N-cadherin, Vimentin, Slug and c-Myc, while decreased that of E-cadherin in BGC-823 and HGC-27 cells. The results of western blot were consistent with that of qRT-PCR (Figure 6B). Moreover, we found that circCCDC66 knockdown inhibited the expression of TGF-β, p-SMAD2 and MMP9 while circCCDC66 overexpression had the opposite effects (Figure 6C). In summary, circCCDC66 could regulate GC progression by regulating c-Myc and TGF-β signaling pathways.

### c-Myc and TGF-β1 knockdown abrogates the promotion of GC cell proliferation, migration, and invasion by circCCDC66

**Figure 7:**
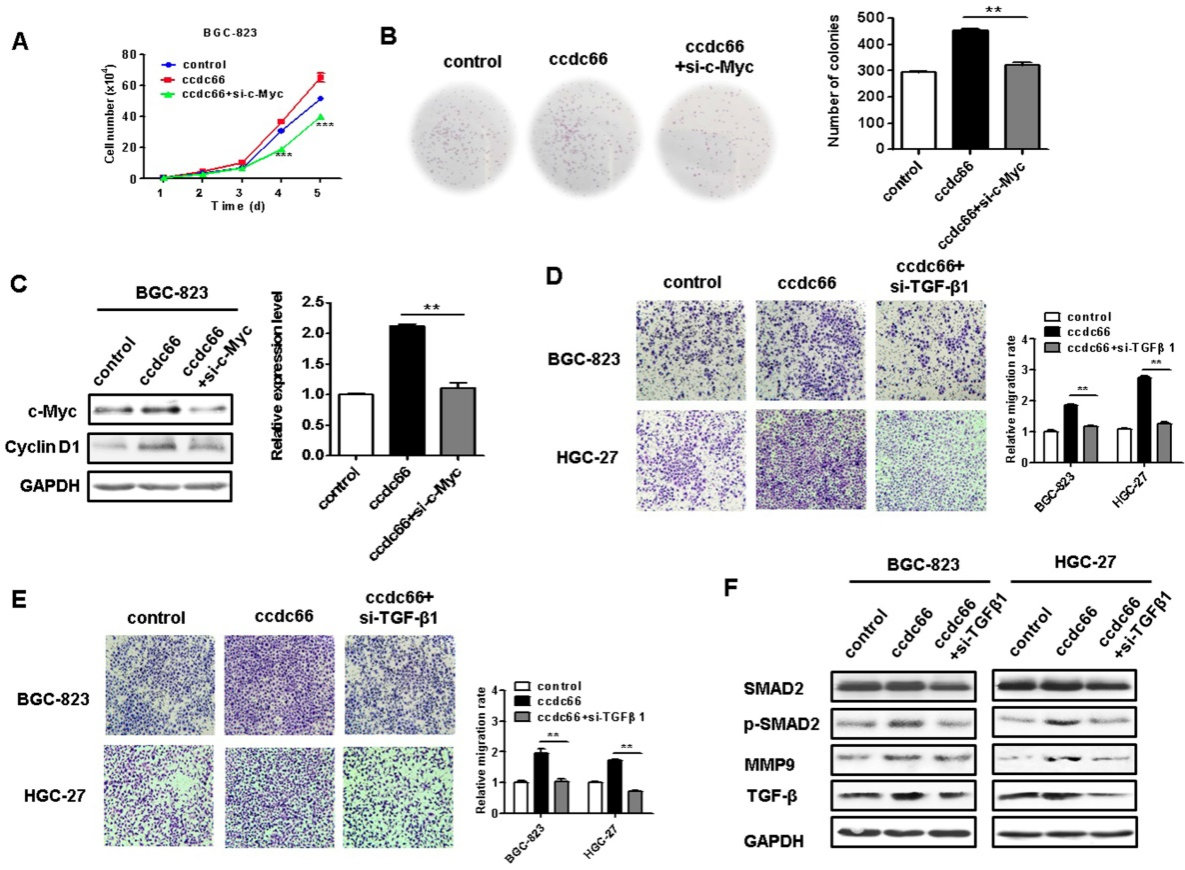
Inhibition of c-Myc and TGF-β1 suppresses the promotion of GC cell proliferation, migration and invasion by circCCDC66. (**A**) Cell growth curves for circCCDC66-overexpressing GC cells with or without c-Myc knockdown. (**B**) The colony formation ability of circCCDC66-overexpressing GC cells with or without c-Myc knockdown. (**C**) Western blot analyses of c-Myc and cyclin D1 expression in circCCDC66-overexpressing GC cells with or without c-Myc knockdown. (**D**) Transwell migration and (**E**) Matrigel invasion assays for the migration and invasion abilities of circCCDC66-overexpressing GC cells with or without TGF-β1 knockdown. (**F**) Western blot analyses of TGF-β, p-SMAD2, and MMP9 expression in circCCDC66-overexpressing GC cells with or without TGF-β1 knockdown. All the experiments were performed in triplicate. *P<0.05, **P<0.01, ***P<0.001, compared to circCCDC66 group.

Since c-Myc is a powerful oncogene in cancer cell proliferation, we knocked down c-Myc in circCCDC66-overexpressing BGC-823 cells to see if it affects GC cell proliferation. We found that c-Myc knockdown remarkably reversed the promoting roles of circCCDC66 overexpression in GC cell proliferation (Figures 7A and 7B). The knockdown of c-Myc also inhibited the upregulation of cyclin D1, a downstream target of c-Myc, in circCCDC66-overexpressing GC cells (Figure 7C). To further demonstrate the importance of TGF-β1 in the oncogenic roles of circCCDC66 in gastric cancer, we knocked down TGF-β1 expression in circCCDC66-overexpressing BGC-823 and HGC-27 cells by using siRNA. Compared with circCCDC66-overexpressing group, TGF-β1 knockdown impaired the promotion of GC cell migration and invasion by circCCDC66 (Figures 7D and 7E). TGF-β1 knockdown also inhibited the upregulation of p-SMAD2 and MMP9 in circCCDC66-overexpressing GC cells (Figure 7F). In summary, circCCDC66 could regulate GC cell proliferation, migration, and invasion by regulating c-Myc and TGF-β signaling pathways.

## Discussion

In recent years, circular RNAs have attracted great interest in cancer research field. Several circRNAs have been discovered to be aberrantly expressed in tumor tissues, plasma, serum and gastric juice of GC patients 30-32. However, the expression pattern and biological function of circCCDC66 in GC have not been revealed. Herein, we reported that circCCDC66 was upregulated in GC tissues and cell lines, and its upregulation was significantly associated with tumor stage and lymphatic metastasis, suggesting a potential of circCCDC66 to be used as a marker for GC diagnosis. We further investigated the effects of circCCDC66 on gastric cancer progression and elucidated the underlying mechanisms. We found that depletion of circCCDC66 suppressed the proliferation, migration and invasion of GC cells but promoted the apoptosis of GC cells. CircCCDC66 overexpression showed the opposite effects. The results of *in vivo* study showed that the tumors in si-circCCDC66 group had smaller volumes and weights than that in si-control group, implicating that circCCDC66 is critically involved in GC progression.

CircRNAs have been identified as critical regulators of gene and protein expression in gastric cancer 33-37. For instance, the expression of circOSBPL10 is upregulated in GC tissues and its level is a prognostic marker of the overall survival and disease-free survival of patients with GC. CircOSBPL10 promotes the growth and metastasis of gastric cancer by regulating miR-136-5p-WNT2 axis 38. The expression of circAKT3 is higher in cisplatin (CDDP)-resistant GC tissues and cells than that in CDDP-sensitive samples. CircAKT3 promotes DNA damage repair and inhibits the apoptosis of GC cells by sponging miR-198 to upregulate PIK3R1 expression 39. CircCCDC66 expression is first discovered to be elevated in colon cancer and is associated with poor prognosis 21. CircCCDC66 controls cell proliferation, migration, and invasion via regulation of a subset of oncogenes. Recently, Wen *et al.* demonstrate that circCCDC66 targets DCX to regulate cell proliferation and migration by sponging miR-488-3p in Hirschsprung's disease 40. Recently, Yang* et al.* suggest that circCCDC66 knockdown attenuates the proliferative and invasive abilities of GC cells by regulating miR-1238-3p/LHX2 axis 41. In consistent with these findings, we reported that circCCDC66 was upregulated in GC tissues and cells and circCCDC66 overexpression promoted while circCCDC66 knockdown inhibited GC cell proliferation, migration and invasion, suggesting that circCCDC66 plays an oncogenic role in GC.

EMT is closely associated with tumor progression and metastasis and is regulated by various signaling pathways 42. The previous studies have shown that circRNAs could induce EMT to promote tumor progression. For example, Li and colleagues demonstrate that cir-ITCH inactivates wnt/β-catenin pathway to inhibit esophageal squamous cell carcinoma 43. Recently, Zhou *et al.* suggest that circRNA_0023642 could promote GC cell migration and invasion through the regulation of EMT 44. In this study, we showed that circCCDC66 knockdown inhibited the expression of TGF-β and inactivated SMAD signaling pathway, thus reversing EMT in GC cells, suggesting that circCCDC66 is an important regulator of EMT in gastric cancer. We also found that circCCDC66 knockdown decreased while circCCDC66 overexpression increased the expression of c-Myc in GC cells. Our results are consistent with that reported by Hsiao *et al.* showing that circCCDC66 could upregulate c-Myc expression by acting as miRNA sponge for miR-33b and miR-93 21. However, the mechanism by which circCCDC66 regulate c-Myc and TGF-β signaling pathways in GC cells is currently unknown and deserves further investigation.

## Conclusions

In summary, circCCDC66 was upregulated in GC tissue and its expression level was significantly associated with tumor progression. CircCCDC66 knockdown inhibited while circCCDC66 overexpression promoted GC cell proliferation, migration and invasion. CircCCDC66 knockdown induced GC cell apoptosis and suppressed tumor growth. CircCCDC66 played oncogenic roles in GC via the activation of c-Myc and TGF-β signaling pathways.

## Supplementary Material

Supplementary figures and tables.

## Abbreviations

CircRNAs: circular RNAs
circCCDC66: circular RNA CCDC66
CRC: colorectal cancer
EMT: epithelial to mesenchymal transition
GC: gastric cancer
HCC: hepatocellular cancer
RBPs: RNA binding proteins
