# Supplementary material for: Circular RNA CCDC66 promotes gastric cancer progression by regulating c-Myc and TGF-β signaling pathways
Source: J Cancer. 2020 Feb 20;11(10):2759–68. doi: 10.7150/jca.37718 (PMC7086271; doi:10.7150/jca.37718)
Supplement: Supplementary file 1 — Supplementary figures and tables. [file jcav11p2759s1.pdf]

Supplementary Table 1. The association between circCCDC66 expression levels and the clinicopathological features.

**Features**	**Number**	**circCCDC66 expression**		**Mean±SD**	***P* value**
		**High**	**Low**		
**Gender**					
Male	47	32	15	5.96±1.94	0.122
Female	23	11	12	6.24±1.59	
**Age, years**					
＜60	18	10	8	6.50±2.24	0.584
≥60	52	33	19	5.90±1.66	
**Tumor size (cm)**					
＜5	35	21	14	5.73±1.74	1
≥5	35	22	13	6.38±1.88	
**Differentiation**					
Moderate	29	17	12	6.08±1.64	0.804
Poor	41	26	15	6.03±1.97	
**Lymphatic metastasis**					
N0	18	6	12	6.17±1.49	0.01
N1-3	52	37	15	6.01±1.94	
**Venous or Perineural invasion**					
Absent	39	22	17	5.90±1.64	0.459
Present	31	21	10	6.24±2.05	
**Invasion depth**					
T1 and T2	5	3	2	5.95±1.47	1
T3 and T4	65	40	25	6.06±1.86	
**TNM stage**					
I and II	19	7	12	6.28±1.52	0.014
III and IV	51	36	15	5.97±1.94	
**Tumor location**					
Antrum	14	8	6	6.44±2.17	0.832
Body	11	8	3	6.44±1.61	
Angulus	5	3	2	4.51±1.41	
Cardia	21	14	7	6.10±1.71	
Others	19	10	9	5.89±1.83	
**CA199***					
Normal	49	30	19	6.41±1.73	0.767
Abnormal	16	11	5	5.29±1.98	

*indicates missing 5 cases.
